# Analysis of collaborative robot technology patent map and research on development trends

**DOI:** 10.1038/s41598-025-14074-0

**Published:** 2025-08-17

**Authors:** Di Zhang, Lihua Liu

**Affiliations:** College of Mechanical and Electronic Engineering, Shanghai Jianqiao University, Shanghai, 201306 People’s Republic of China

**Keywords:** Collaborative robots, Patent analysis, Technological innovation, Geographical distribution, PatSnap, Mechanical engineering, Information technology

## Abstract

This study conducts a systematic analysis of global patents in the field of collaborative robot technology based on applications and type of industry. The data analyzed derives from the PatSnap database covering 30,425 patents from 2006 to 2025, using explicit keywords such as “collaborative robot” to ensure specificity, with procedural filters and manual curation applied to maximize relevance and accuracy. The research focuses on patent application trends, technological hotspots, geographical distribution, as well as applicant and inventor analyses, providing a comprehensive overview of the innovation landscape and development trends in this domain. The findings indicate that collaborative robot technology has undergone three distinct phases: the initial emergence period, a phase of rapid growth, and a subsequent slowdown, and it is now transitioning into a stage of mature development. This apparent recent slowdown is significantly influenced by the standard 18-month lag in patent publication, meaning current data for the latest years is incomplete. Invention patents dominate the field, reflecting a high level of technological innovation. From a geographical perspective, the United States and China serve as the primary global innovation hubs, with their patent application volumes significantly surpassing those of other countries. In terms of applicants, multinational corporations such as Qualcomm, Intel, BRIGHT DATA LTD, and NVIDIA lead in patent filings, while emerging enterprises have demonstrated notable growth in patent applications, injecting new vitality into the industry. Furthermore, through patent citation analysis and market value assessment, this study identifies high-value patents and key technological areas, offering valuable insights for future technological advancements and market strategies. Based on these findings, the study proposes recommendations to further promote the sustainable development of collaborative robot technology, including strengthening technological innovation, optimizing market positioning, fostering emerging enterprises and talent, enhancing international cooperation, and aligning with policy directions.

## Introduction

Collaborative robots (cobots) are advanced robotic systems engineered to operate safely and efficiently alongside humans or other robots in shared workspaces. Unlike traditional industrial robots, which typically function in segregated, caged areas, cobots are primarily intended to enhance, rather than wholly replace, human capabilities. They achieve this integration by combining the strength, precision, and endurance of machines with the flexibility, problem-solving abilities, and cognitive skills of human workers.

Cobots are equipped with advanced capabilities including perception, real-time decision-making, sophisticated control, and interactive functions, enabling them to dynamically adapt to changes in the work environment. Crucially, while ensuring human operator safety, cobots contribute to enhanced production efficiency, operational flexibility, and precision in task execution.

In recent years, the application of collaborative robots has expanded significantly across various sectors, notably in industry, healthcare, and education. In industrial settings, cobots are primarily deployed to undertake repetitive, ergonomically demanding, or high-precision tasks, thereby freeing human workers to engage in more complex and value-added activities. For instance, in the automotive and electronics industries, cobots are widely used for screw-driving, pick-and-place operations, and quality inspection. In logistics, they aid in sorting and packing procedures, while in the medical field, they function as assistants in surgical procedures and rehabilitation sessions.

The introduction of cobots collaborating with humans or other robots has led to marked improvements in production efficiency, workplace safety, and operational flexibility^1,2^. Notable advancements have been achieved in perception, control, and human–robot interaction technologies, enabling the execution of increasingly complex tasks^3^. In manufacturing, cobots are extensively utilized for assembly, logistics, and warehousing applications; through optimized path planning and advanced obstacle avoidance, they support efficient and safe operations^4–6^. In healthcare, they are used for surgical assistance and rehabilitation training, improving the quality of medical services through precise control algorithms and tactile feedback mechanisms^7–9^. In education, cobots function as interactive teaching aids, facilitating the understanding of complex concepts and fostering students’ interest in technology^10,11^.

With ongoing technological advancements, cobots have realized substantial improvements in sensory perception, control strategies, and human–machine interfaces, allowing them to undertake ever more sophisticated tasks. Nevertheless, several key questions remain: What are the prevailing global trends in collaborative robot technology? What technological gaps exist between China and developed countries in this field? What are the prospective directions for the future development of collaborative robotic systems? Patents, as a crucial indicator of technological innovation, provide valuable insights into the trajectory and developmental trends of emerging technologies. According to statistics from the World Intellectual Property Organization (WIPO), over 90% of global technological innovations are documented in patent literature. Therefore, analyzing patent application trends, geographic distribution, International Patent Classification (IPC) codes, high-frequency keywords, and patterns of patent collaboration can offer a comprehensive understanding of the current state and future directions of collaborative robot technology. Patents, as a key medium for technological innovation, can reveal the trajectory and trends of technological development. According to the statistics from the World Intellectual Property Organization (WIPO), over 90% of global technological innovations achievements are recorded in the form of patent literature. Therefore, analyzing patent application trends, geographic distribution, International Patent Classification (IPC), high-frequency keywords, and patent cooperation networks from the perspective of patent literature is of significant importance for gaining a comprehensive understanding of the current status and future trends in the field of collaborative robot technology.

Therefore, analyzing the patent application trends, regional distribution, International Patent Classification (IPC), high-frequency keywords, and patent cooperation network of collaborative robot technology from the perspective of patent literature is of great significance for a comprehensive understanding of the current development status and future trends in this field^12,13^.

## Research methods and data sources

### Research methods

Patent mapping is an analytical tool that extracts, filters, classifies, and synthesizes patent literature, presenting the analysis results in graphical and tabular forms, thus providing a navigational function similar to a map for the technological field^14^. Patent map analysis plays a crucial role in areas such as industrial competitive intelligence and corporate strategic decision-making. In this study, the patent mapping method is employed to deeply analyze patent literature, reveal the key technological features in the field of collaborative robot technology, and predict future development trends^13^.

### Data sources

The data analyzed in this study is derived exclusively from the PatSnap patent database, which complies records from 172 national and regional patent offices, covering more than 190 million patent documents. The database provides comprehensive coverage and is recognized for its high reliability. PatSnap serves as a globally recognized platform for intellectual property analytics and is regarded as a highly accurate source for patent research. This study focuses on the patent data related to collaborative robots in the “PatSnap” database. The search period spanned was set from 2006 to 2025, with the search keywords being “collaborative robots” or “collaborative robot”. The search query—restricted to patents explicitly referencing the terms “collaborative robot” and equivalents—was designed to ensure statistical data closely reflect strictly Cobot-related innovations, thereby minimizing the inclusion of irrelevant technologies.

To maximize data reliability, manual curation and a relevancy check were performed on the aggregated dataset. Nonetheless, inherent limitations remain, including inconsistencies in patent terminology, classification differences among national patent offices, and the multidisciplinary nature of robotics innovations. Therefore, the reported figures should be interpreted as robust, high-confidence estimates rather than absolute totals.

Although this keyword-based approach represents a standard methodology in patentometrics, it inherently retrieves patents from various multi-disciplinary fields (e.g., AI, IoT, medical devices) that incorporate cobot technology. provides a realistic representation of the cross-disciplinary innovation ecosystem associated with collaborative robotics. The accuracy of the dataset reflects the patents captured by this defined and reproducible search strategy. The data retrieval and download were completed on April 7, 2025, yielding 30,425 relevant patent records, providing a solid data foundation for the subsequent analysis.

Data extraction, analysis, and visualization in this study were facilitated by the PatSnap platform, which offers proprietary big data analysis, citation tracking, and patent valuation algorithms. Additional data processing and trend analysis were conducted using Microsoft Excel, while visualizations were generated in Python (matplotlib/seaborn), in accordance with standard bibliometric practices.

## Patent application trends and regional distribution

### Trend of patent applications

**Fig. 1:**
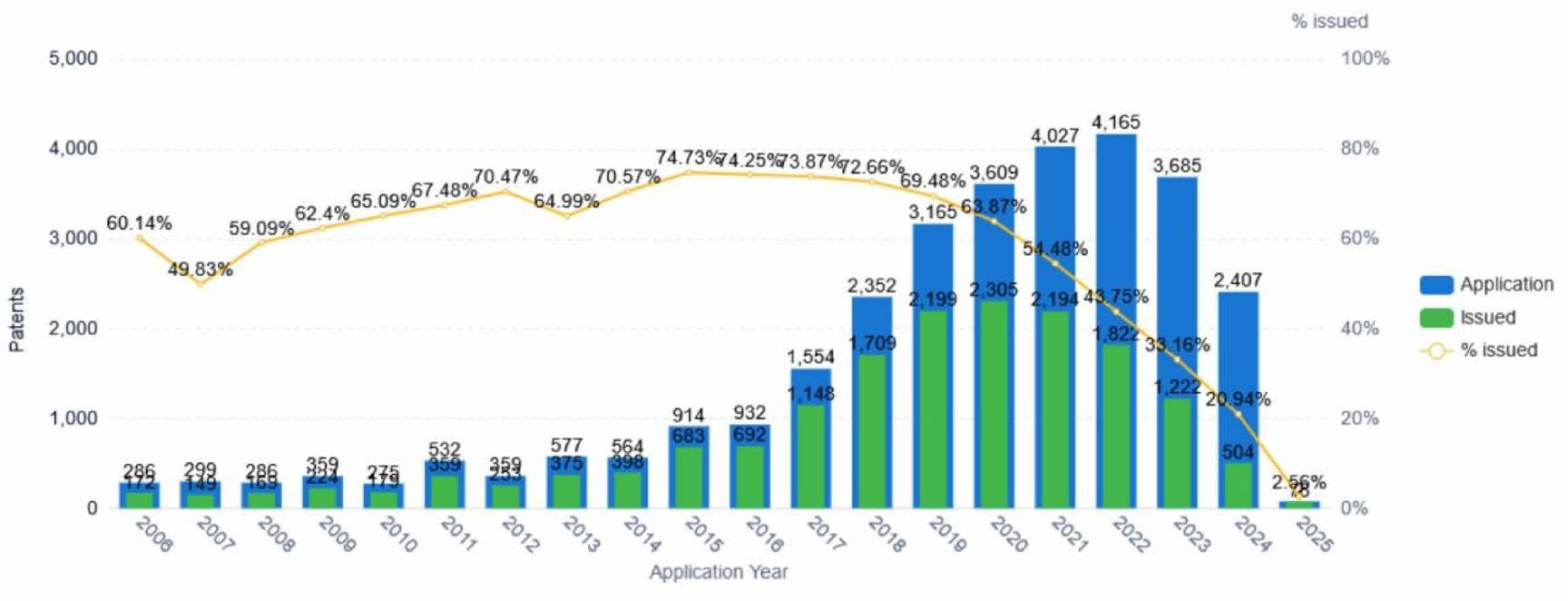
Global annual patent applications for collaborative robot technology (2006–2025).

As shown in Fig. 1, a systematic analysis of the global annual trend in collaborative robotics patent applications provides a comprehensive understanding of the technological within this field. From 2006 to 2025, the global development of collaborative robot technology can be divided into three distinct stages. Firstly, during the nascent period (2006–2016), the number of collaborative robot patent applications demonstrated slow yet steady growth, indicating that the technology was still in the exploratory phase. During this period, numerous enterprises and research institutions entered the field sequentially, fostering initial technological development and laying a foundation for subsequent breakthroughs. Next, in the period of rapid growth (2017–2022), the number of collaborative robot patent applications increased sharply, with the market experiencing explosive growth. This rapid development was primarily driven by breakthroughs in technological innovation, expanding market demand, government policy and capital support, declining manufacturing costs, and enhanced user-friendliness. Furthermore, since market competition had not yet stabilized, companies raised their R&D investments, further advancing technological progress and broadening the scope of applications.

However, from 2023 to 2025, the number of patent applications entered a declining phase, reflecting the gradual maturation of the market. During this period, collaborative robot technology faced several challenges, including limitations in perception, understanding, and decision-making capabilities, safety concerns, optimization of human–machine interaction design, and cost control^15^. Notably, technological bottlenecks remain particularly prominent, requiring further improvement to accommodate increasingly complex application environments. The sharp decline in newly issued collaborative robot patents after 2022 can be attributed to the following factors :

1. A natural slowdown in core technological innovation as the field matures;
2. A strategic shift by companies from patent quantity to portfolio quality, consolidating filings and concentrating investments on commercialization;
3. A lag in public disclosure due to the standard 18–24 month embargo prior to publication;
4. More stringent patent examination standards and overlapping legal rejections, which further reduce the rate of new issuances.

Additionally, global economic uncertainty may have also affected R&D budget allocations.

It is important to note that, due to the time lag between patent application and publication, as well as the lengthy approval process, the number of granted patents between 2006 and 2021 continued to increase year by year. Therefore, although the number of patent applications has decreased in recent years, granted patent data should be considered in a comprehensive manner to accurately assess the overall technological development in this field.

### Patent types and legal status

**Fig. 2:**
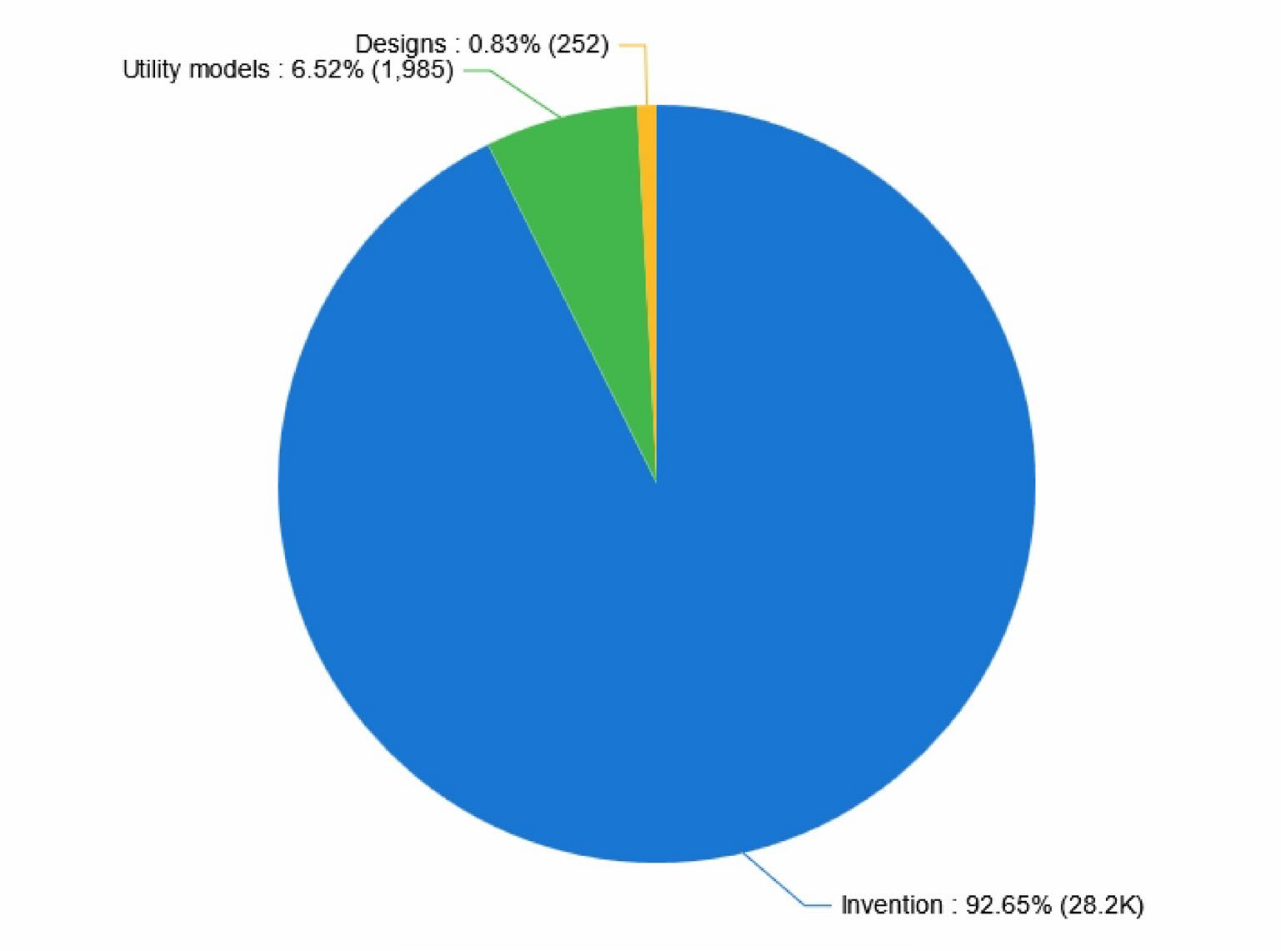
Statistics of patent types in collaborative robotics.

As shown in Fig. 2, with the patent applications in collaborative robot technology, the proportion of invention patents is the highest, accounting for 92.65%, while the proportions of utility models and design patents are 6.52% and 0.83%, respectively. This distribution pattern indicates that technological innovation predominates in the field of collaborative robots. Although design patents are rare, they protect distinctive ornamental, ergonomic, or user-interface aspects specific to Cobots—such as specialized housings for medical or educational robots—which contribute to product differentiation in regulated or consumer-focused markets. Although less prevalent, these design patents play an important role in sectors (such as health care and consumer robotics) where product form, safety, visual appeal, and unique design are critical determinants of market acceptance and user experience. In contrast, utility models and invention patents address core functional, structural, algorithmic, and control system innovations [minor wording adjustment for precision]. This division demonstrates that Cobot development is highly technology-driven, with an emphasis on functional advances, although design innovation remains essential for end-user adoption and competitive differentiation (e.g., ergonomic medical robots, accessible educational robots).

**Fig. 3:**
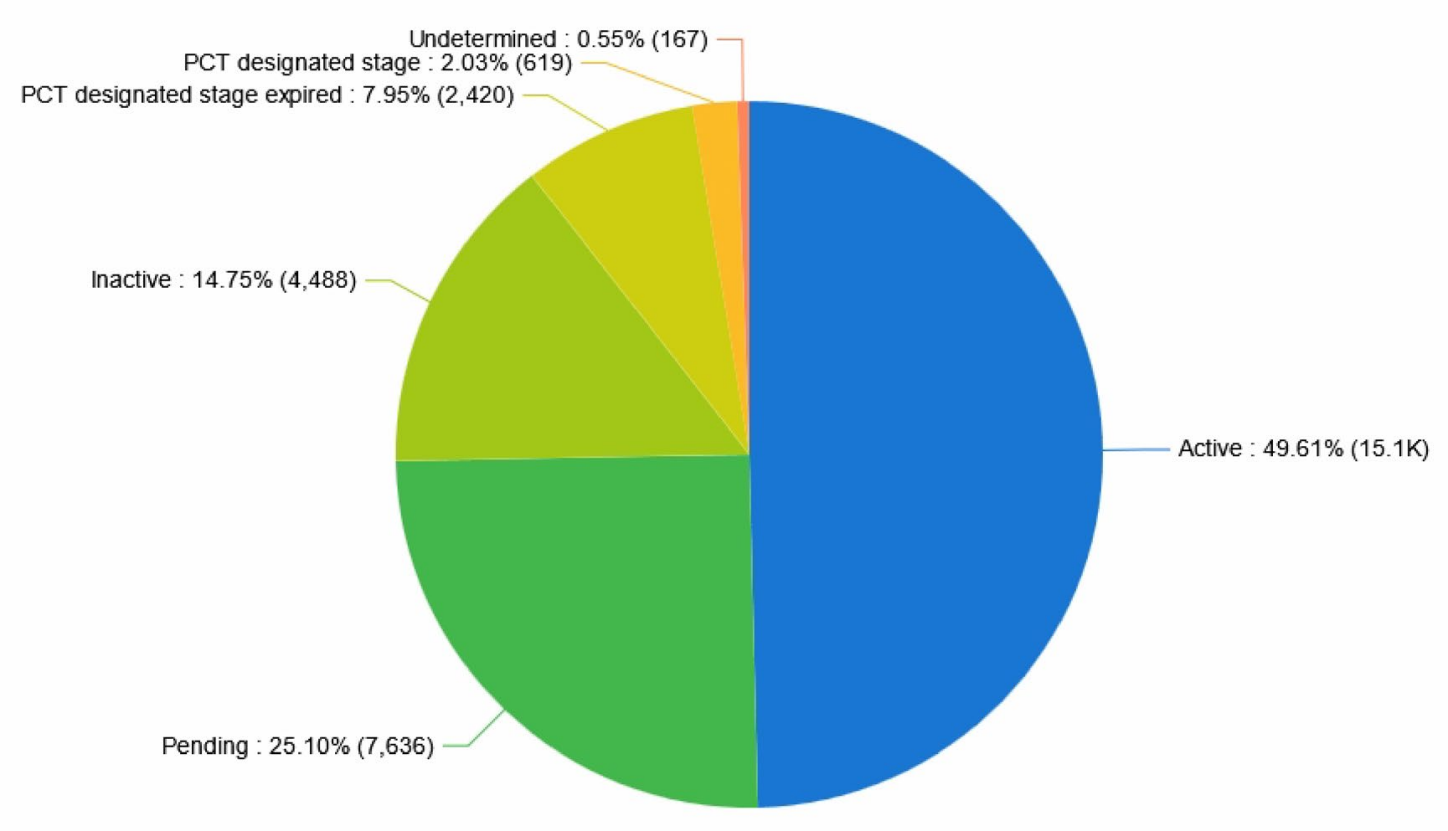
Statistics of legal status of collaborative robotics patents.

Figure 3 shows the distribution of the legal status of patents related to collaborative robots, the proportion of valid patents is the highest, reaching 49.61%, indicating that enterprises in this field maintain strong innovative capacity in technology research and development. This high percentage of granted and active patents further suggests a relatively high quality and successful prosecution rate among filed applications, though a more detailed country-specific analysis of grant rates falls beyond the scope of this study.

### Global patent distribution

**Fig. 4:**
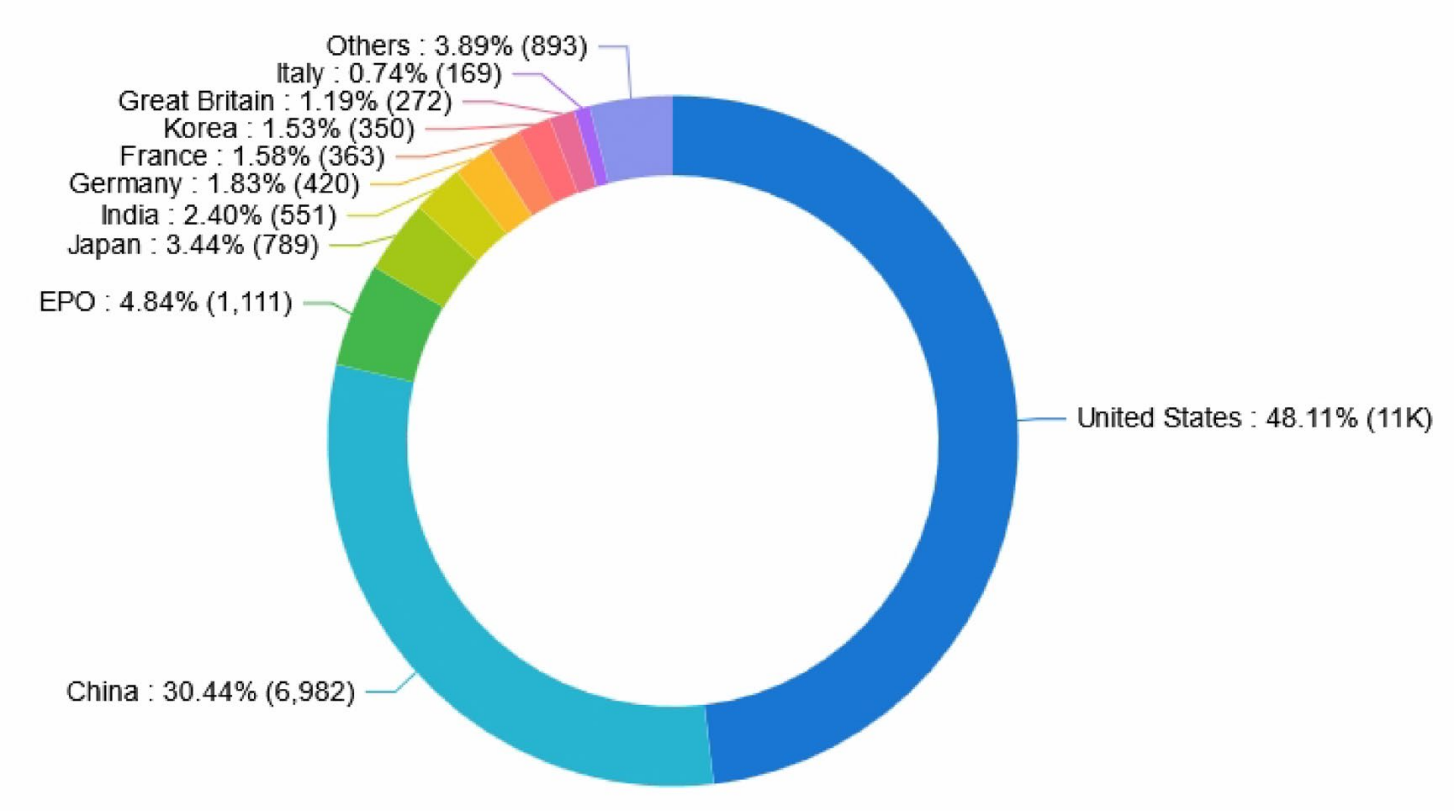
Distribution of patent quantities by country and region worldwide.

Figure 4 presents the distribution of global collaborative robotics patents by country and region from 2006 to 2025. For analysis, each patent was counted once by deduplicating records based on the publicly available text of each application, using the most recent publication date for data aggregation. The results show that the top ten countries/regions in terms of collaborative robotics patent applications.

At the national level, the United States leads in collaborative robotics patent filings, followed by China, Japan, and the European Patent Office (EPO). However, the EPO’s statistics represent regional patent protection within Europe, encompassing filings not only from European applicants but also from U.S., Japanese, and Chinese companies; hence, this number does not solely reflect indigenous European innovation. Germany, despite being a recognized leader in industrial automation and a key user of collaborative robots, registers a relatively low patent application share (1.83%). This relatively modest filing volume can be attributed to both Germany’s heavy reliance on the EPO route and the likelihood that German innovation in this domain is concentrated more on system integration, process optimization, and application-specific solutions—areas that might be safeguarded as trade secrets or through process patents, rather than by foundational hardware patents for collaborative robots. Similarly, the large number of filings at the EPO underlines the strategic importance of the European market, encouraging innovators worldwide to seek protection in Europe. Consequently, this figure does not imply that 4.84% of collaborative robotics innovations originated exclusively from other European countries not listed individually.

### Patent quantity ranking by province and municipality in China

**Fig. 5:**
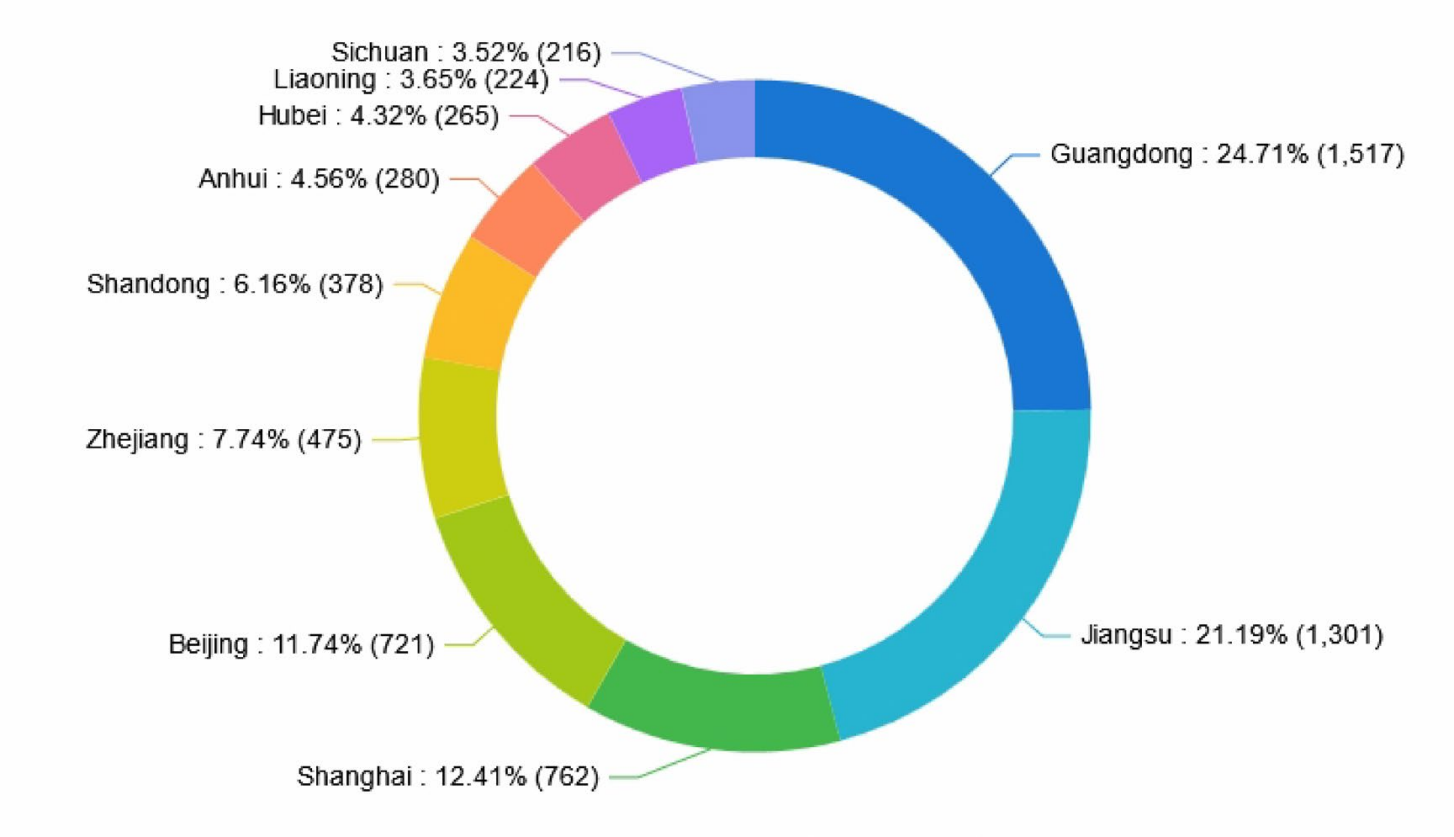
Distribution of patent quantities by province and municipality in China.

Figure 5 depicts the distribution of collaborative robotics technology patents across domestic provinces and municipalities. According to Fig. 5, Guangdong leads China’s collaborative robot patent landscape, with the majority of applications concentrated in manufacturing automation, electronics assembly, and logistics. Jiangsu and Shanghai stand out for applications primarily related to automobile manufacturing, semiconductors, and smart warehousing, while Beijing is distinguished by its strengths in health care robots, educational robots, and AI-driven human–robot interaction. Key industries driving patent applications in these regions are:

- Guangdong: Electronics, home appliances, and consumer goods manufacturing.
- Jiangsu: Automotive, logistics, and equipment manufacturing.
- Shanghai: Smart manufacturing, biomedical, AI, and ICT industries.
- Beijing: Health care, education, and research institutes.

Sectors such as electronics, automotive, and health care predominate, owing to significant investment in automation and supportive government policies. The total number of patents filed by these four provinces and municipalities constitutes approximately 70.02% of the national patent applications. This indicates that these regions possess strong substantial capabilities in the research and development of collaborative robotics technology. Additionally, regions such as Zhejiang, Shandong, Anhui, Hubei, Liaoning, and Sichuan are also actively fostering scientific talent and enhancing innovation. Collectively, their patent filings represent approximately 29.98% of national applications.

#### Ranking of the target market trends of patents

**Fig. 6:**
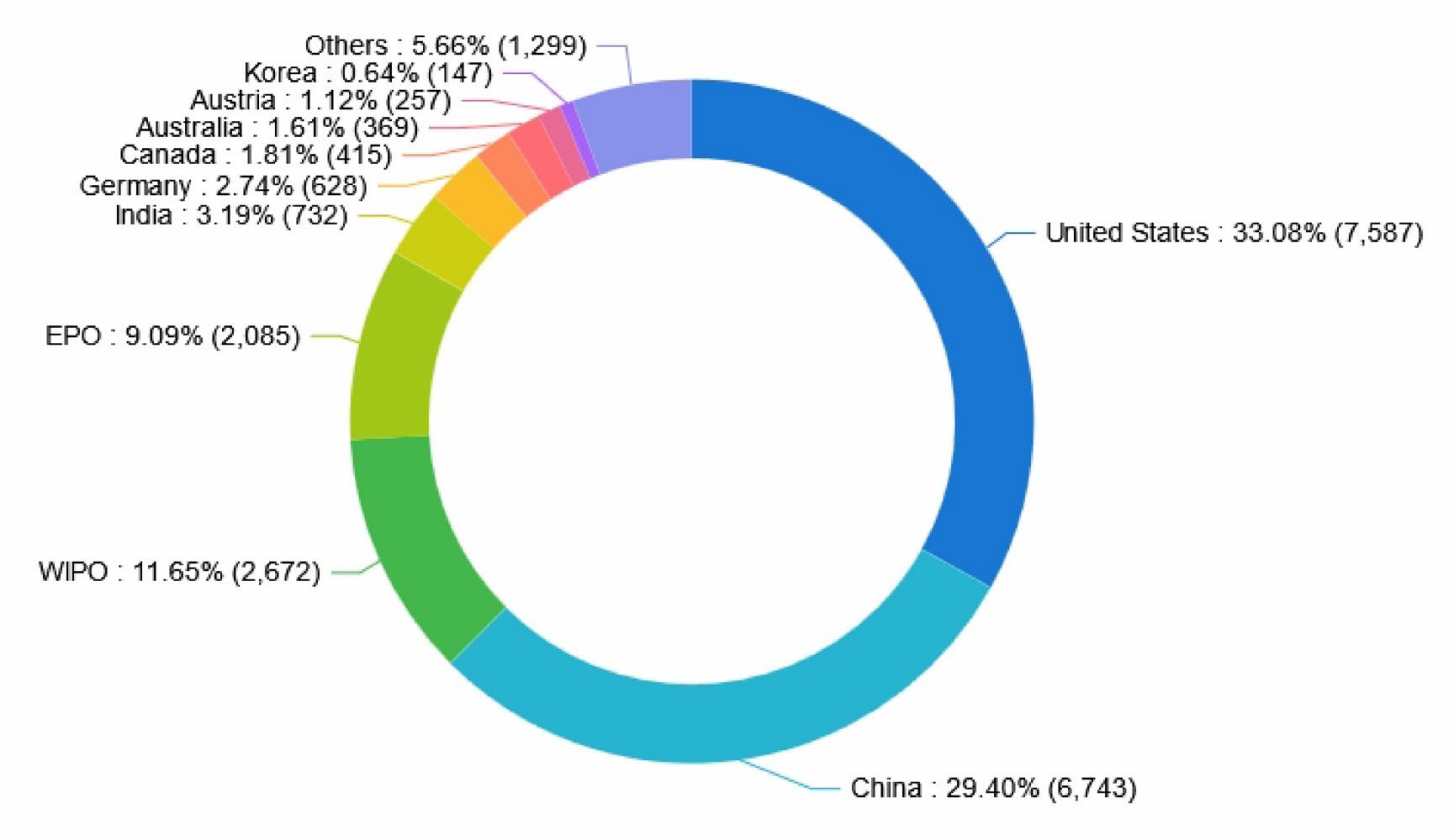
Ranking of patent target markets by country/region.

Figure 6 illustrates the ranking of target markets for collaborative robotics patents by country/region. The top ten countries/regions are as follows: the United States (33.08%), China (29.40%), the World Intellectual Property Organization (WIPO) (11.65%), EPO (9.09%), India (3.19%), Germany (2.74%), Canada (1.81%), Australia (1.61%), Austria (1.12%), and Korea (0.64%). Together, these account for 94.34% of total global patent applications. Patent technologies related to collaborative robotics are primarily concentrated in China, the United States, and Europe. The number of patent applications to a certain extent reflects the level of interest in these target markets.These findings can assist companies in determining which countries or regions should be prioritized within their technology strategies and in identifying markets that remain under-addressed, thereby revealing new potential growth opportunities.

## Analysis of collaborative robotics patent applicants and inventors

### Applicant ranking analysis

**Fig. 7:**
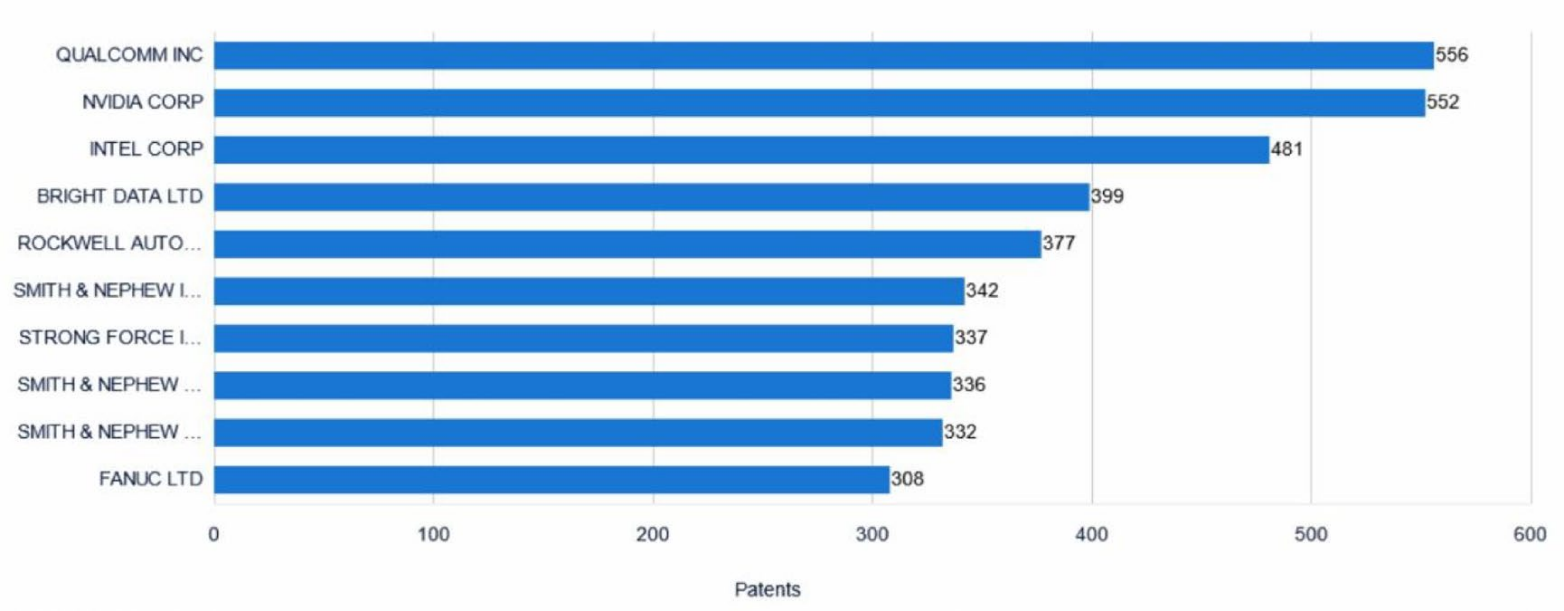
Ranking of the top 10 patent applicants.

Figure 7 presents the top ten companies by global collaborative robotics patent application volume. These companies exhibit significant technological innovation as well as robust research and development capabilities within their respective fields. Qualcomm Incorporated leads the ranking with 556 patent applications, representing 1.83% of the total. As a leading American semiconductor and telecommunications equipment company, Qualcomm focuses on the development of wireless communication technologies, with its technical expertise highly regarded within the industry. Closely following is NVIDIA Corporation, with 552 patent applications (1.81%). This American semiconductor company is renowned for its production of graphics processing units (GPUs) and related computing technologies, with products widely used in gaming, artificial intelligence, and other fields. Intel Corporation ranks third with 481 patent applications (1.58%).This multinational semiconductor company is renowned for producing computer processors (CPUs), and its technological innovations continually drive the development of the computing industry. BRIGHT DATA LTD ranks fourth, holding 399 patent applications (1.31%). Headquartered in Israel, the company is a global leader in network data platforms, focusing on data collection, web scraping technologies, and network data solutions, and provides high-quality data support to various industries.NOKIA AUTOMATION CONTROL TECHNOLOGY CO., LTD. ranks fifth, holding 377 patent applications (1.24%). and focuses on research an development in automation control technologies, PLC instruments, safety automation systems, electronic and electrical products, and sensors.

Additionally, Smith & Nephew and its subsidiary, Smith & Nephew Orthopaedics Inc., round out the top ten, holding 342 and 336 patent applications, respectively (1.12% and 1.10%). These companies focus on the medical device sector, offering innovative solutions for wound management, orthopedics, endoscopic surgery, and plastic surgery. STRONG FORCE IOT PORTFOLIO 2016 LLC holds 337 patent applications (1.11%) and is dedicated to IoT technology development and related applications. SMITH & NEPHEW ASIA PACIFIC PTE LTD ranks ninth with 332 patent applications (1.09%) and specializes in orthopedics, neurosurgery, and surgical instruments as a global medical device company.

FANUC Corporation also makes the top ten, holding 308 patent applications, tying with Smith & Nephew Orthopaedics Inc. for seventh place (1.01%). This Japanese industrial automation company is known for its CNC machine tools and industrial robots. Xerox Singapore ranks tenth with 302 patent applications (1.06%) and is also a global medical device company specializing in orthopedics, neurosurgery, and surgical instruments.

The global ranking of the top ten patent applicants in collaborative robotics demonstrates that these companies excel in technological innovation and R&D. They not only possess strong technical capabilities in their respective domains but also propel industry-wide development through continual innovation. Their patent portfolios encompass both traditional sectors—such as semiconductors, telecommunications equipment, and medical devices—and emerging fields like IoT and automation control technologies. These innovations and applications will help lay a solid foundation for the future development of collaborative robotics.

### Analysis of major applicants’ technological distribution

**Fig. 8:**
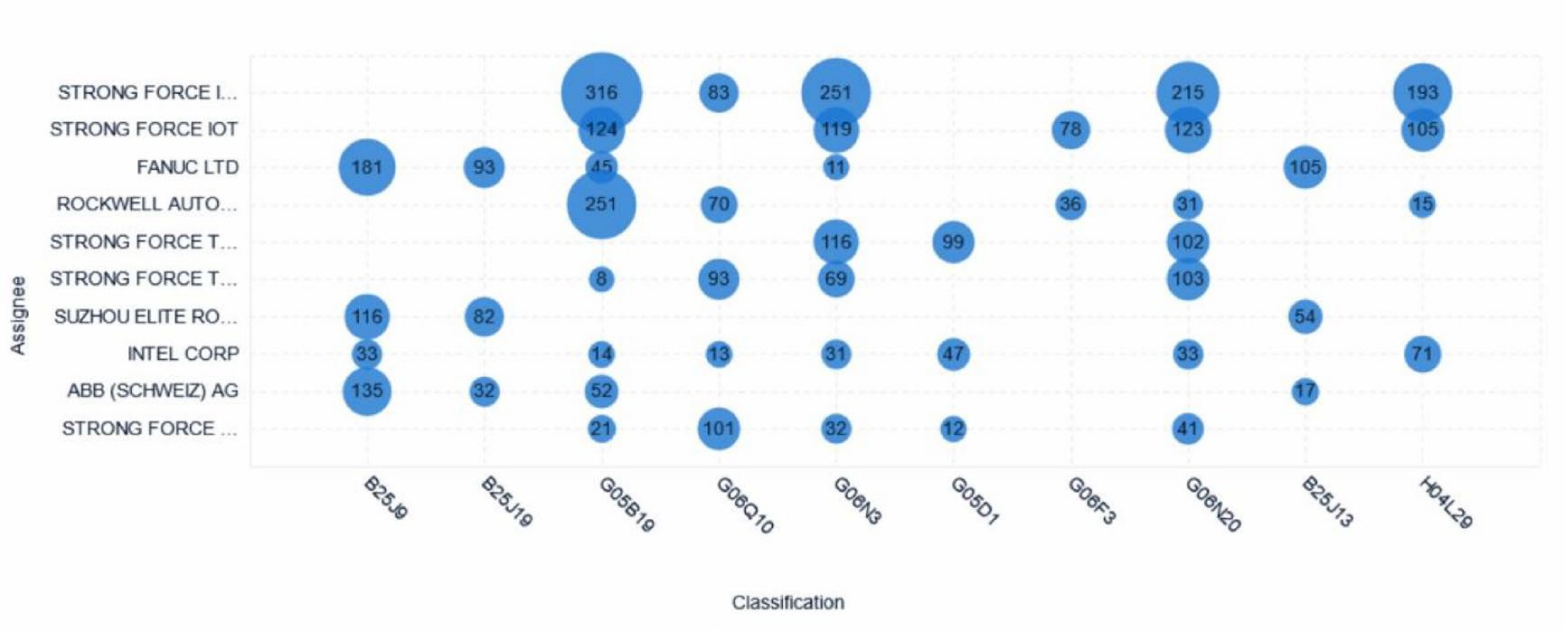
Bubble chart of technological distribution of major patent applicants.

Figure 8 presents a visual representation of the technological distribution of the top ten patent applicants in the global collaborative robotics field. The horizontal axis indicates technology classification numbers, while the vertical axis lists the applicants. The size of the bubbles intuitively reflects the number of patent applications each applicant has made in different technological branches, thereby revealing their specific research directions and key focus areas in collaborative robotics technology.

From the figure, it can be observed that STRONG FORCE IOT PORTFOLIO 2016 LLC demonstrates a broad distribution of patent applications across multiple technology classification numbers, reflecting its comprehensive R&D capabilities in the collaborative robotics field. This suggests that the company is extensively involved across various branches of collaborative robotics technology, showcasing diverse and holistic technical strengths. In contrast, Xerox Singapore, Smith & Nephew, and Smith & Nephew Orthopaedics Inc. Each have a significant concentration of patent applications in specific technology classification numbers. This reflects their in-depth research and specialized focus in particular technological areas, suggesting they may posses unique technological advantages in these fields.

Rockwell Automation Control Technology Co., Ltd. and BRIGHT DATA LTD display a more scattered application distribution, indicating their exploration of diverse aspects of collaborative robotics technology and efforts to achieve breakthroughs across different technical branches.

Although Qualcomm, Intel, NVIDIA, and FANUC have relatively fewer patent applications overall, their concentrated filings in certain specific classification numbers show their focus and depth in these areas, suggesting they may possess strong competitiveness in these particular technological branches.

This analysis further reveals that the activity and significance of different technological branches vary, providing important insights into industry trends and the future direction of technological development.

### Application trends of major applicants

**Fig. 9:**
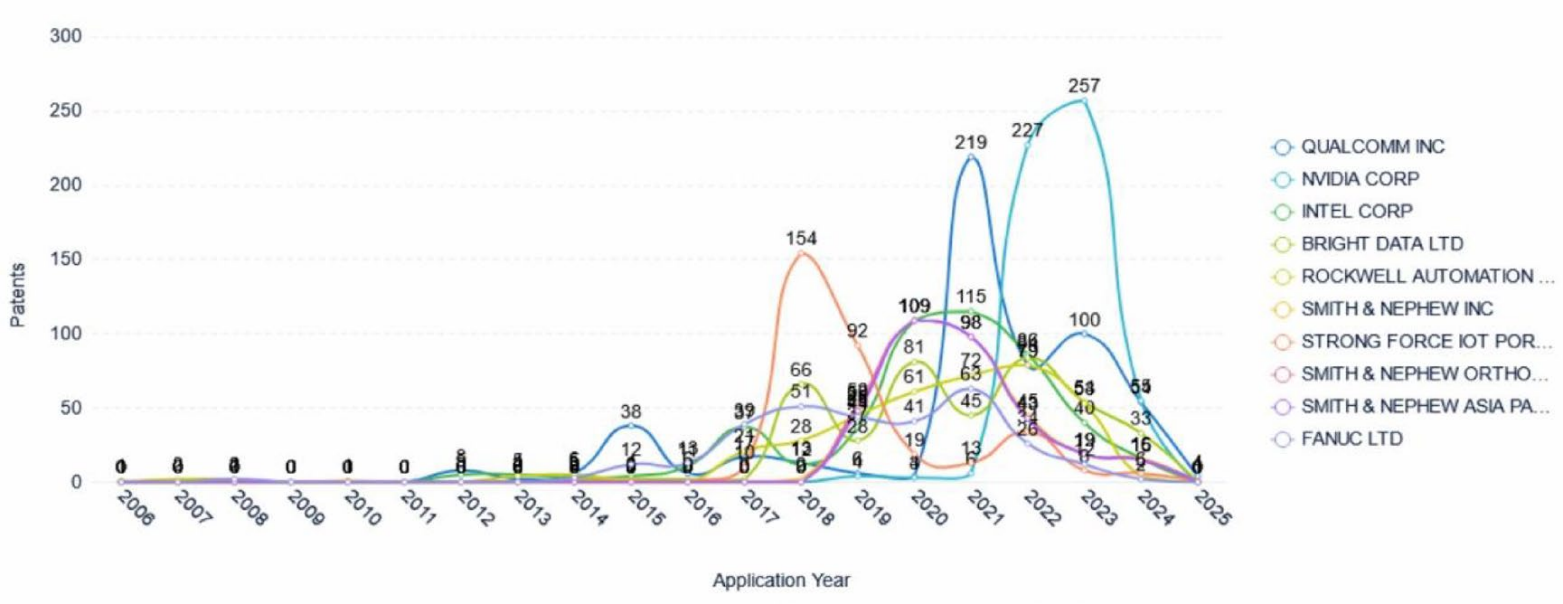
Patent application trends of major applicants.

Figure 9 depicts the changing trends in patent applications by major applicants in the field of collaborative robot technology. NVIDIA Corporation showed relatively high application volumes in 2022 and 2023, with 227 and 257 patents filed, respectively. This trend indicates NVIDIA’s concentrated investment in collaborative robot during these years, likely aligned with its business development strategy and market positioning. Qualcomm Incorporated displayed significant peaks in patent applications in 2021 and 2023, with 219 and 100 patents filed, respectively. This suggests that Qualcomm committed substantial R&D resources to collaborative robotics in these periods, possibly aiming to capture emerging market opportunities or respond to technological shifts. STRONG FORCE IOT PORTFOLIO 2016 LLC experienced elevated application volumes in 2018 and 2019, filing 154 and 92 patents, respectively. This peak may have resulted from major breakthroughs in collaborative robotics technology or strategic adjustments, underscoring the company’s strong innovation capabilities in this sector. Smith & Nephew and Intel generally maintained stable levels of patent applications, but both exhibited an uptick in 2020 and 2021. This pattern reflects their sustained commitment and investment in collaborative robotics technology, possibly in response to evolving market demands or technological challenges.

### Inventor ranking analysis

**Fig. 10:**
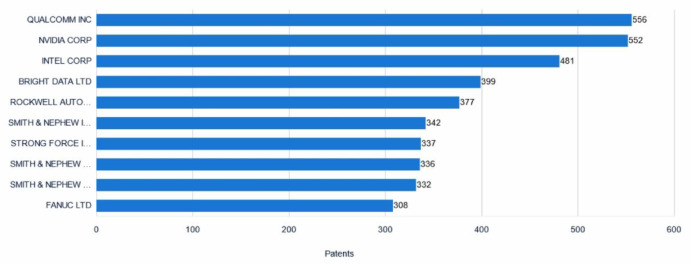
Inventor ranking analysis chart.

In the field of collaborative robotics technology, the number of patents held by key inventors serves as a key indicator of their contributions and expertise. As shown in Fig. 10, several outstanding inventors stand out based on their patent filings. CELLA, CHARLES S. leads by a substantial margin with 753 patents, establishing himself as a highly influential figure in this area. MOGUCKIN, JEFF R. and DUFFY, JR., GERA. follow with 429 and 417 patents, respectively, demonstrating their significant contributions. SHRIBMAN, DERRY and VILENSKI, OFER each hold 385 patents, tying for fourth place and marking them as key contributors. They are followed by VAN DER AUWERA, S. and KARCZEWICZ, MA., who hold 340 and 339 patents, respectively. Other notable inventors include RAMASUBRAMONIA with 325 patents, DESAI, MEHUL with 307 patents, and CARDNO, ANDREW with 252 patents, all of whom occupy significant positions in the field.

These data not only highlight the varying levels of contributions among inventors in collaborative robotics technology but also provide valuable insights for companies seeking top talent in specific technological areas. For organizations aiming to recruit leading inventors, these top-ranked individuals represent key targets of interest. By gaining a deeper understanding of their research directions and achievements, companies can more precisely identify the necessary talent, thereby maintaining a competitive edge in the field.

## Key technology analysis

### Patent technology field analysis

**Fig. 11:**
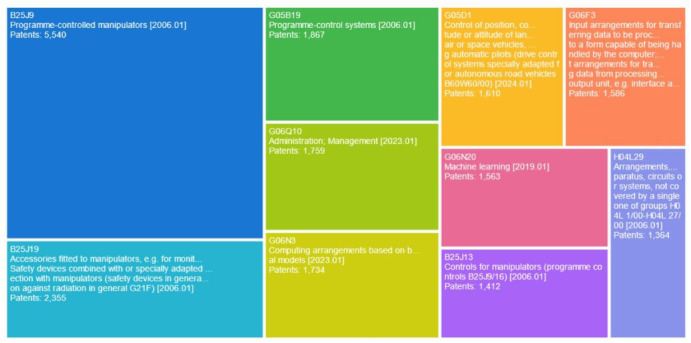
Technological composition.

Figure 11 illustrates the distribution of key technological fields in collaborative robotics, based on patent counts. From the analysis of Fig. 11, it becomes apparent that patent activity is primarily concentrated in the following areas, ordered by the number of patents granted: program-controlled manipulators (B25J9), accessories fitted to manipulators (B25J19), program-control systems (G05B19), administrative management (G06Q10), computing arrangements based on biological models (G06N3), autopilot control systems (G05D1), data input/output devices or circuits (G06F3), machine learning (G06N20), Manipulator control systems (B25J13) and controls for manipulators(B25J13).

This analysis reveals a multi-layered innovation ecosystem, which can be summarized by the following key findings:The analysis reveals a multi-layered innovation ecosystem with the following key observations:

1. Dominant Core Technologies: Program-controlled manipulators (B25J9) stand out as the central hub of innovation, accumulating 5,540 patents. This reflects both technological maturity- given the classification’s establishment since 2006—and sustained research and development activity, underscoring their foundational role in robotic hardware advancement. Accessories fitted to manipulators (B25J19) hold the second position with 2355 patents, indicating vigorous complementary innovation, especially in sensor integration and safety systems, as specified in the classification description.
2. Control System Innovations:The notable representation of both program-control systems (G05B19: 1867 patents) and autopilot control systems (G05D1: 1610 patents) emphasizes an industry-wide focus on precision control technologies. In particular, the 2024 update to the G05D1 classification suggests ongoing advancements in autonomous navigation applications.
3. Integration of Advanced Computing: Computing arrangements inspired by biological models (G06N3:1734 patents) and machine learning (G06N20: 1563 patents) highlight significant convergence between neuroscience-inspired computing and AI-driven robotics. The 2019 introduction of the machine learning classification reflects the relatively recent integration of these techniques within the field.
4. Human–Machine Interfaces: Substantial progress in data input/output systems (G06F3: 1586 patents) points to active development in tactile feedback technologies and sensor integration, as indicated by the classification’s emphasis on “transferring data to be processed… handled by the computer.”
5. Operational Infrastructure: Administrative management systems (G06Q10: 1759 patents), updated as recently as 2023, underscore the growing focus on robotic workflow optimization and enterprise-level integration, suggesting increasing maturity in industrial deployment scenarios.
6. Emerging Specializations: Manipulator control systems (B25J13: 1412 patents) and controls for manipulators (H04L29: 1364 patents) form critical support structures, notably, the latter represents a fragmented classification (“not covered by single groups”), which hints at ongoing innovation in distributed robotics communication systems.

Technological Maturity Insights: The establishment of core manipulator technology classifications (B25J9, B25J19) in 2006 contrast with the recent updates in autonomous control (G05D1:2024) and management systems (G06Q10:2023). This indicates an industry shift from mechanical innovation towards the integration of intelligent system. This evolution is reflected in patent volumes: while established hardware domains maintain their leading position, computational methods such as AI and machine learning are demonstrating remarkable potential for rapid growth.

### Application trends of patent technology branches

**Fig. 12:**
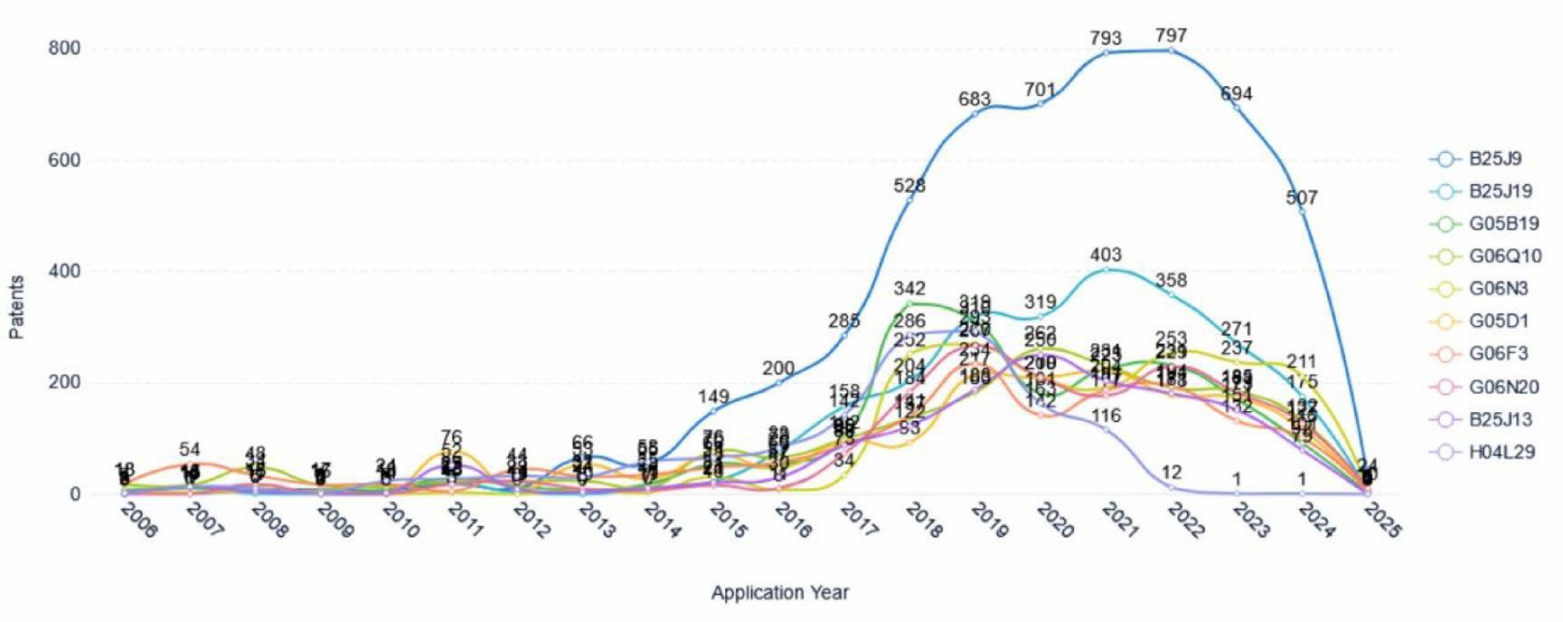
Technology branch application trends.

Figure 12 illustrates the annual patent application trends for major technical branches in collaborative robotics from 2006 to 2025. The data reveals several distinct phases across this time frame. Initially, from 2006 to approximately 2015, the number of patent applications across these technical branches remained relatively low and stable. A marked inflection point occurred around 2016–2017, signifying the onset of a rapid growth phase in application activity across most categories-indicative intensified research and development efforts within the field. This surge culminated in a peak period spanning roughly 2019–2022. Notably, Programme-controlled manipulators (B25J9) exhibited the most pronounced increase in patent filings, reaching a near-peak of 793 applications in 2021 and achieving an absolute maximum of 797 applications in 2022. Accessories fitted to manipulators (B25J19) also experienced considerable growth, peaking at 403 applications in 2021. Simultaneously, other technical branches such as Programme-control systems (G05B19), administration management (G06Q10), and Computing arrangements based on biological models (G06N3) also recorded significant increases, with peak application numbers generally occurring between 2019 and 2020. Other tracked branches followed similar, albeit less pronounced, growth trajectories during this period. Following this peak, however, the data indicate a sharp and widespread decline in patent application numbers beginning in 2023, with the downward trend continuing steeply through 2024 and 2025 across all major technical branches illustrated. This pronounced downturn could potentially be attributed to factors such as market saturation, cyclical adjustment in technological investment, consolidation in the industry, or possibly delays in the publication of the most recent patent data. In summary, the patent application trends for technical branches of collaborative robotics demonstrate a period of intense activity and investment peaking around 2019–2022, followed by a significant recent contraction as reflected in the data up to 2025. This underscores the highly dynamic and evolving nature of innovation within the field.

### Geographic distribution of key technical branches

**Fig. 13:**
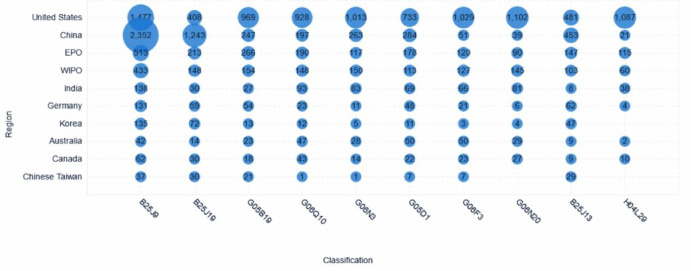
Geographical distribution of key technology branches.

Figure 13 provides a comparative analysis of patent application volumes in specific technical branches relevant to collaborative robots, delineated by geographic region or filing authority. The vertical axis represents the region or authority, while the horizontal axis details pertinent International Patent Classification (IPC) codes. The magnitude of patent activity at each region-classification intersection is represented by the size of the bubble, with the precise count indicated numerically. The data reveal a distinct concentration of patenting activity within the United States and China, albeit with markedly different technological emphases. China demonstrates clear leadership in classifications B25J9 (Manipulators not otherwise provided for; 2352 patents) and B25J19 (Programme-controlled manipulators; 1243 patents), indicating a significant focus on core mechanical architectures and programmed control of robots. Conversely, the United States exhibits a broader technological footprint: while it holds a substantial portfolio in B25J9 (1477 patents), the US leads significantly across across most other classifications presented. This includes high patent volumes in areas potentially associated with advanced control, sensing, AI, networking, and specific applications, such as G05B19 (Programme control systems; 969 patents), G06Q10 (Administration/management; 928 patents), G06N3 (Biologically-inspired computer systems/AI; 1,013 patents), G05D1 (Control of non-electric variables; 733 patents), G06F3 (Data input arrangements; 1029 patents), G06N20 (Machine learning; 1102 patents), B25J13 (Manipulator control systems/procedures; 481 patents), and H04L29 (Network arrangements; 1087 patents).Patent filings through the European Patent Office (EPO) and the World Intellectual Property Organization (WIPO) represent the next tier of activity, highlighting considerable international interest and multi-jurisdictional patent strategies, however, these regions generally lag behind the US and China in volume within specific classifications. Other individual regions shown, including Germany, Korea, India, Australia, Canada, and Chinese Taiwan-exhibit comparatively lower patent counts across these technical branches.

Overall, these patterns suggests ongoing, but less voluminous, research and development efforts in collaborative robotics within these territories relative to the leading nations. In summary, the patent landscape for these key collaborative robot technologies is dominated by the United States and China, each displaying distinct areas of technological concentration. The US exhibits leadership across a broad spectrum of advanced control, AI, and networking-related classifications, while China maintains a commanding lead in the core mechanical structures and programmed operation categories. Other regions contribute to the field, but at a demonstrably lower scale of patenting intensity in these specific technical domains.

### Global patent keyword cloud analysis

To elucidate dominant themes and technological focal points within recent collaborative robotics innovation, a word cloud visualization (Fig. 15) was generated from keywords extracted from the latest 5,000 relevant patent documents. In this visualization, keyword frequency is expressed via font size, with larger terms indicating higher prevalence within the patent literature^16^.

**Fig. 14:**
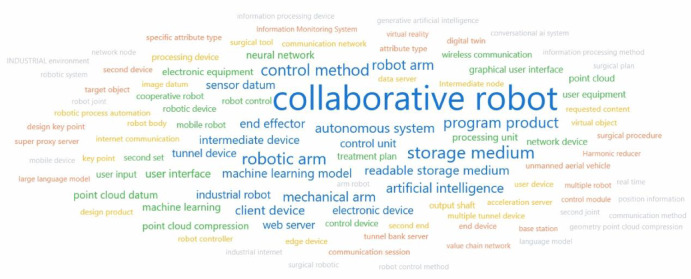
2006–2025 Collaborative robot innovation word cloud.

As shown ed in Fig. 14, collaborative robot occupies the central and most prominent position, anchoring the analysis. Several key technological domains emerge with high frequency. Notably, terms associated with core robotic hardware such as “robot arm,” “mechanical arm,” and “end effector” are significant, as are fundamental concepts like “control method” and “robot control.” Strong emphasis on intelligence and data processing is evident through the prominence of terms such as “artificial intelligence,” “machine learning,” and “neural network.” Data handling and perception concepts, particularly “point cloud,” “sensor datum,” and “point cloud compression,” are likewise conspicuous. Furthermore, terms associated with software, computing infrastructure, and interaction, including “storage medium,” “program product,” “processing unit,” “user interface,” and “graphical user interface-are also well represented. The presence of secondary keywords such as “autonomous system,” “digital twin,” “wireless communication,” “mobile robot,” and “industrial robot” further delineates the technological landscape. Less frequent, but nevertheless visible, terms like “surgical tool,” “large language model,” and “edge device” may point to specialized application domains or emerging technological trajectories within the collaborative robotics domain.

In summary, this keyword frequency analysis derived from recent patent data underscores a strong focus on the integration of artificial intelligence and machine learning with core robotic components (arms, control systems), advanced sensing modalities (especially point clouds), and the requisite computing infrastructure. These dominant themes reflect the principal technological drivers shaping the current state and near-term development directions of collaborative robots.

## High-value patent analysis

High-value patents refer to those characterized by substantial technical merit, high-quality documentation, robust rights stability, and forward-looking potential. Such patents can influence industry development trajectories, demonstrate significant market value, and serve as critical drivers of innovation^17^. Using patent citation rankings and patent market value rankings from platforms such as Patsnap, the distribution of high-value patents in the collaborative robot field is analyzed and compared. This section aims to identify the most influential and commercially significant innovations by employing these two distinct metrics.Patent citation analysis enables identification of a patent’s applicability and impact with its technological field^18^.

### Patent citation analysis

**Fig. 15:**
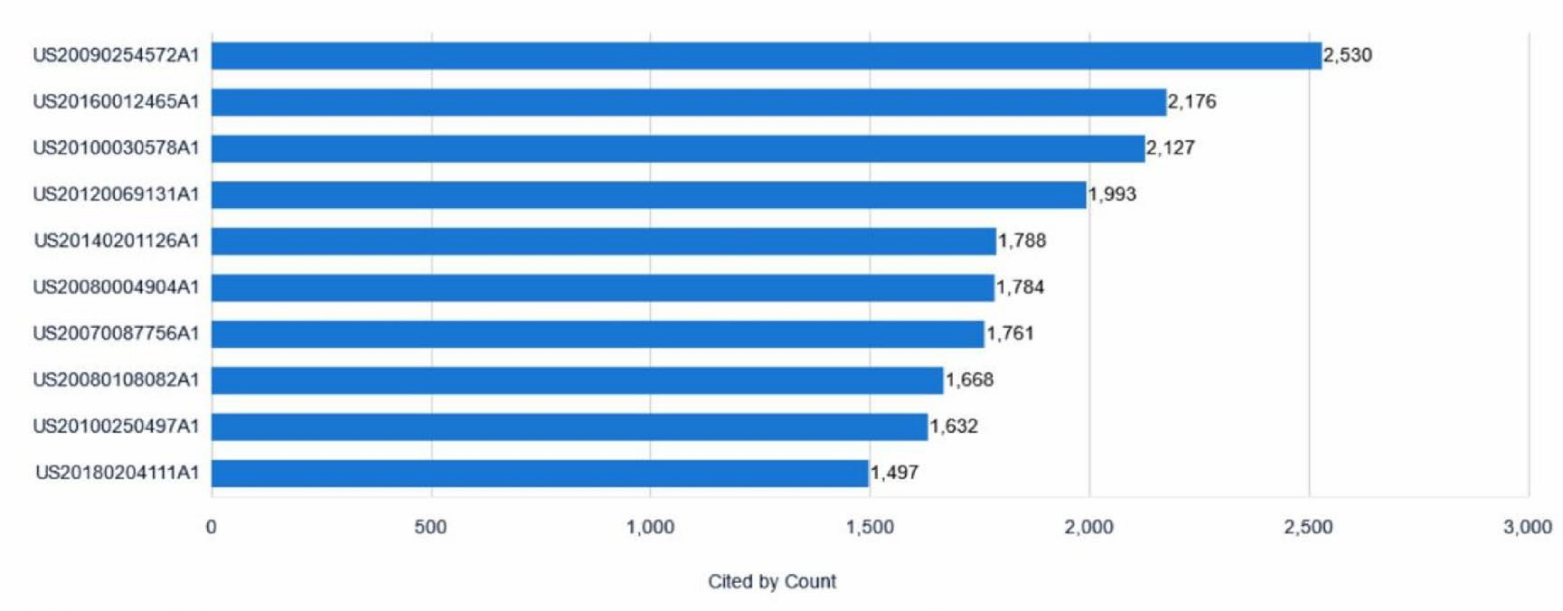
Top 10 most cited collaborative robot patents.

Figure 15 presents a ranking of the ten most patent documents globally within collaborative robot technology; the ranking is based on forward citations-a widely accepted metric for assessing a patent’s technological impact and foundational importance. The vertical axis lists specific patent publication numbers, and the horizontal axis quantifies the number of citations for each patent.

The analysis reveals that US20090254572A1 is the most frequently cited in this cohort, with 2,530 citations. This substantial citation count signals its considerable influence and foundational role within the field. Other highly cited documents include US20160012465A1 (2176 citations), US20100030578A1 (2127 citations), and US20120069131A1 (1993 citations). High citation rates are generally indicative of a patent’s technological significance and its role in stimulating subsequent innovation and development. A salient finding from Fig. 15 is the geographic origin of these highly influential patents. Notably, all ten documents listed are United States patent publications. This observation suggests a strong concentration of the most impactful innovations, as measured by citation frequency within this specific top-tier group, originating from or filed within the United States. This concentration underscores the prominent role of US-based innovation in shaping the trajectory of wearable medical device technology, based on the analyzed citation data.

### Patent market value analysis

Assessment of patent market value provides additional insights into the potential commercial significance of inventions within a given technological field. Patents of high market value frequently indicate important technological advancements and strong market impact. In this study, the Patsnap patent analysis platform was utilized to identify patents with high estimated market values relevant to collaborative robotics. The ranking is based on Patsnap’s proprietary valuation algorithm. Patsnap employs a proprietary valuation system informed by the Failure Mode and Effects Analysis (FMEA) methodology, incorporating multi-dimensional data validation and refined algorithms, supplemented by machine learning for continuous optimization^18^. It should be noted that this valuation system is restricted to invention applications, granted inventions, and utility models, and does not include design patents in its scope^18,19^.

Table 1 presents the top 10 patents with the highest estimated market values as determined by the Patsnap analysis within the context of this study. To facilitate interpretation, a column categorizing the primary application domain of each patent has been added. Notably, the highest estimated valuation of $12,800,000 is attributed to US 12218795B2 (“Internet of things,” assigned to Intel Corp.). The second-ranked patent, EP3342554B1 (“Robot system and operation method thereof”; Kawasaki Jukogyo KK) has an estimated value of $11,700,000. Several other patents in the top ten also exhibit values exceeding $9,000,000, including US20240257594A1 (Federal Express Corp US; apparatus for logistics operations, $10,990,000), EP4383228A3 (Nike Innovate CV; intelligent electronic footwear, $9,700,000), and US11138809B2 (Microsoft Technology Licensing LLC; method for providing an object in virtual space, $9,180,000). Several other patents in the top 10 also exhibit values exceeding $9,000,000, including US20240257594A1 (Federal Express Corp US; Apparatus for logistics operations, $10,990,000), EP4383228A3 (Nike Innovate CV; Intelligent electronic footwear, $9,700,000), and US11138809B2 (Microsoft Technology Licensing LLC; Method for providing object in virtual space, $9,180,000).

**Table 1:** Top 10 highest-market-value patents in collaborative robotics.

Rank	Publication number	Title	Applicant	Value (USD)	Application domain/industry
1	US12218795B2	Internet of things	INTEL CORP	$12,800,000	IoT / Enabling technology
2	EP3342554B1	Robot system and operation method thereof	KAWASAKI JUKOGYO KK	$11,700,000	Core Robotics device
3	US20240257594A1	Apparatus, Systems, and Methods for Performing a Dispatched Logistics Operation for a Deliverable Item from a Hold-at-Location Logistics Facility Using a Modular Autonomous Bot Apparatus Assembly, a Dispatch Server and an Enhanced Remotely Actuated Logistics Receptacle Apparatus	FEDERAL EXPRESS CORP US	$10,990,000	Logistics Automation
4	EP3846981B1	A robot cleaner and a controlling method for the same	LG ELECTRONICS INC	$9,870,000	Robot cleaner
5	EP4383228A3	Intelligent electronic footwear and control logic for automated infrastructure-based pedestrian tracking	NIKE INNOVATE CV	$9,700,000	Medical/Wearable tech
6	US20240171820A1	Recommending media programs based on media program popularity	GOOGLE LLC	$9,600,000	Software/Virtual environments
7	EP4418057A3	Methods and systems for the industrial internet of things	STRONG FORCE IOT PORTFOLIO 2016 LLC	$9,350,000	Control and regulation system
8	US11138809B2	Method and system for providing an object in virtual or semi-virtual space based on a user characteristic	MICROSOFT TECHNOLOGY LICENSING LLC	$9,180,000	Software/Virtual environments
9	EP3662296A4	Detecting location within a network	IVANI	$9,030,000	Artificial intelligence
10	US12261712B2	Managing and selecting proxy devices by multiple servers	BRIGHT DATA LTD	$9,030,000	Communcations technology

Analysis of the patent titles and assignees presented in Table 1 reveals considerable technological diversity. While core robotics patents are present (e.g., Kawasaki), the list is dominated by high-value patents in technologies that enable or complement collaborative systems, such as the Internet of Things (IoT), logistics automation, consumer electronics (footwear), data processing, virtual environments, and network management. This finding suggests that enabling technologies that interact with collaborative robots are perceived as highly valuable. Geographically, U.S.-based assignees (Intel, FedEx, Google, Microsoft) predominate, but significant representation from Japan (Kawasaki), South Korea (LG Electronics, potentially), and Israel (Bright Data) is also observed. Notably, no China-based assignees appear among the ten highest-valued patents according to the methods employed in this analysis.

### Patent map analysis

**Fig. 16:**
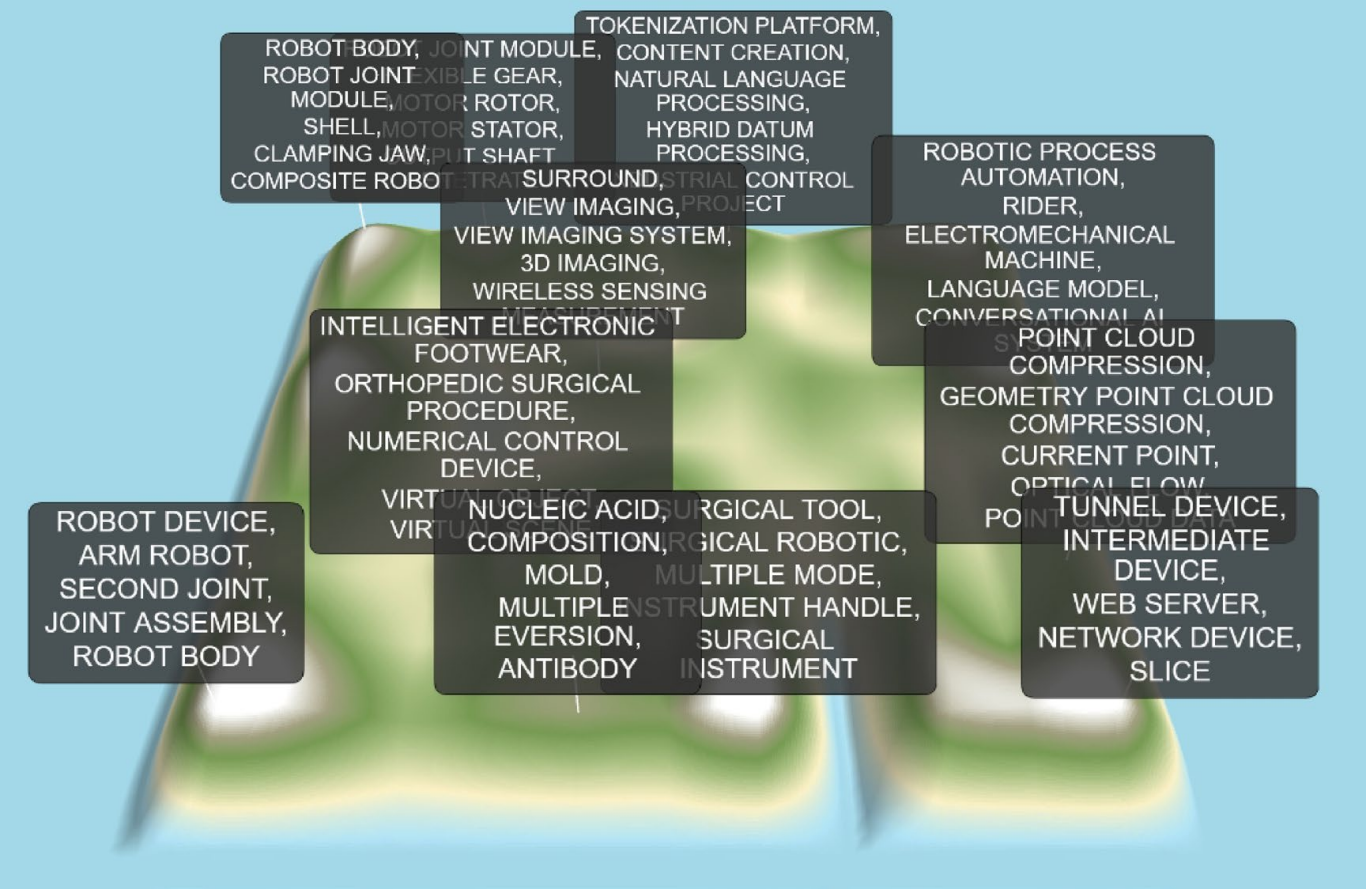
Global collaborative robot technology patent landscape.

This study employs the patent map generation tool integrated within the Patsnap patent database to analyze patents related to collaborative robots. The generated patent map for globally-effective invention patents is displayed in Fig. 16. In the map, bright white areas indicate the highest concentration of patent documents, representing the largest volume of patent applications for those technical themes and representing R&D hot spots. Black areas indicate relatively low patent density. Additionally, the distance between different different thematic regions suggest their degree of technological correlation^17^.

The analysis has been structured to provide a clearer explanation of the clusters identified within this patent landscape (Fig. 16). The description now systematically addresses the major technological themes:

1. *Core Robotic Mechanics*: Core Robotic Mechanics: The largest and most central cluster includes keywords such as “ROBOT BODY,” “JOINT MODULE,” “ROBOT JOINT,” and “ARM ROBOT,” highlighting foundational R&D in core mechanical structures and robotic components.
2. *Perception and Sensing Systems*: Clusters featuring “POINT CLOUD COMPRESSION,” “3D IMAGING,” and “WIRELESS SENSING” emphasize the centrality of environmental perception and interpretation technologies.
3. *Artificial Intelligence and Control*: Clusters for “NATURAL LANGUAGE PROCESSING,” “ROBOTIC PROCESS AUTOMATION,” and “LANGUAGE MODEL” illustrate the integration of AI to facilitate more autonomous and intuitive cobots.
4. *Application-Specific Domains*: Dedicated clusters for, e.g., surgical robotics—with keywords such as “SURGICAL TOOL,” “SURGICAL ROBOTIC,” and “ORTHOPEDIC SURGICAL PROCEDURE”—demonstrate the field’s specialization, particularly in next-generation medical device innovation that enhances collaborative capabilities.
5. *Networking and Digital Infrastructure*: Smaller, distinct clusters associated with “WEB SERVER” and “NETWORK DEVICE” underscore the importance of connectivity and data management for coordinated fleet operation and smart factory integration.

In summary, the patent landscape map illustrates a multifaceted technological field. While foundational work on robot mechanics (joints, bodies, arms) remains central, there are distinct, high-intensity clusters of innovation in areas crucial for advanced collaborative systems: AI, advanced sensing, data processing, networking, and high-value applications domains such as surgical robotics. The distribution and clustering of these themes provide a topographical perspective on R&D priorities within the analyzed collaborative robot patent corpus.

**Fig. 17:**
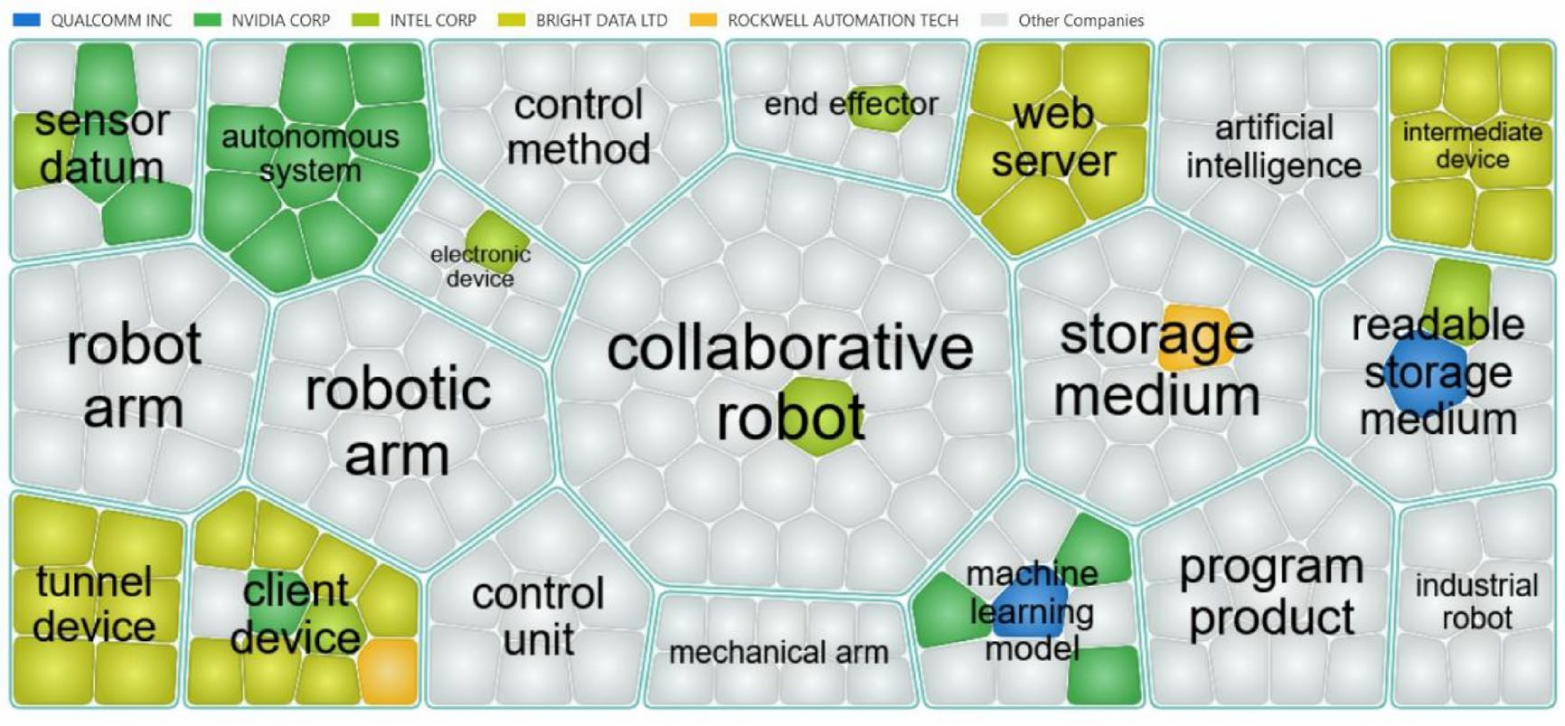
Patent keyword coverage and technological focus map of major companies in collaborative robot technology.

Figure 17 further visualizes the distribution of patent keywords among leading companies in the collaborative robot technology sector, offering insights into each company’s technological focus. The selection is based on the latest 5,000 patent records, ensuring timeliness and relevance. In the figure, grid cells of different colors indicate the patent coverage of major companies across collaborative clusters. This domain map not only displays the distribution of patent keywords for major companies in the collaborative robot technology field but also helps to intuitively differentiate the technological focus and areas of strength of different companies. This is highly significant for understanding industry development trends, grasping the technological frontier, and formulating competitive strategies.

## Conclusions and recommendations

### Conclusions

This study systematically analyzes the global patent landscape for collaborative robot technology and drew the following conclusions:

1. Technology Maturity: Collaborative robot technology has undergone an initial nascent phase, rapid growth, and a recent deceleration, now entering a stage of maturity. Patent application numbers surged from 2017 to 2022 but have declined since 2023, a drop attributed primarily to the 18–24 month patent publication lag, which limits data completeness for the most recent years.
2. Invention Patent Dominance:Invention patents comprise up to 93.33% of filings, far exceeding utility models (5.95%) and design patents (0.72%), indicating that technological innovation—rather than aesthetic or incremental change—remains the primary driver in this field.
3. Global Leaders: The United States and China are established global leaders in collaborative robot technology, with patent filings vastly outpacing other nations. Europe (via the EPO) is positioned as a key market, while regions like Germany focus more on application and integration than foundational patent production.
4. Corporate Patent Strategies: Large multinational corporations, including Qualcomm, Intel, and NVIDIA, are the primary innovators and patent holders in the field. At the same time, an increasing number of emerging domestic companies are actively establishing patent portfolios, leading to a more diversified industrial landscape.
5. High-Value Patents: Analyses of citation and market value reveal that high-value patents predominantly originate in the United States and Europe, especially in areas involving robot system architecture, intelligent control, sensing, and human–machine interaction. In contrast, China has room to further enhance the quality, number, and global influence of its high-value patents.

In summary, collaborative robot technology is now at a relatively mature stage globally, with the patent competition landscape clearly dominated by the United States and China. The future of technological innovation in this field hinges on improving patent quality, optimizing global patent strategies, and fostering technological breakthroughs through international collaboration.

### Recommendations

Based on the above conclusions, the following recommendations are proposed:

Strengthen technological innovation: Enterprises and research institutions should increase investment in core technologies such as perception, control, and human–robot interaction to overcome existing technical bottlenecks.

1. Optimize market positioning: Companies should define their market position based on technological strengths and focus on specific application domains to develop unique competitive advantages.
2. Foster emerging enterprises and talent: Governments and industry organizations should promote the growth of emerging enterprises and cultivate new talent pools for the collaborative robotics field.
3. Enhance international cooperation: Collaborative efforts and exchanges between countries and regions should be strengthened to jointly advance the development and application of collaborative robot technology.
4. Align with policy directions: Enterprises should closely monitor national and regional policy developments, leveraging governmental support to accelerate innovation and market expansion.

In summary, the future development of collaborative robot technology requires synergistic promotion of basic research, optimal patent strategies, international cooperation, and industrial application. By strengthening innovation capacity, elevating patent quality, and deepening global cooperation, collaborative robot technology is poised to achieve broader industrial deployment, thereby providing critical impetus to intelligent manufacturing and the advancement of future industrial automation.

## Data Availability

The datasets used and analysed during the current study available from the corresponding author on reasonable request.
